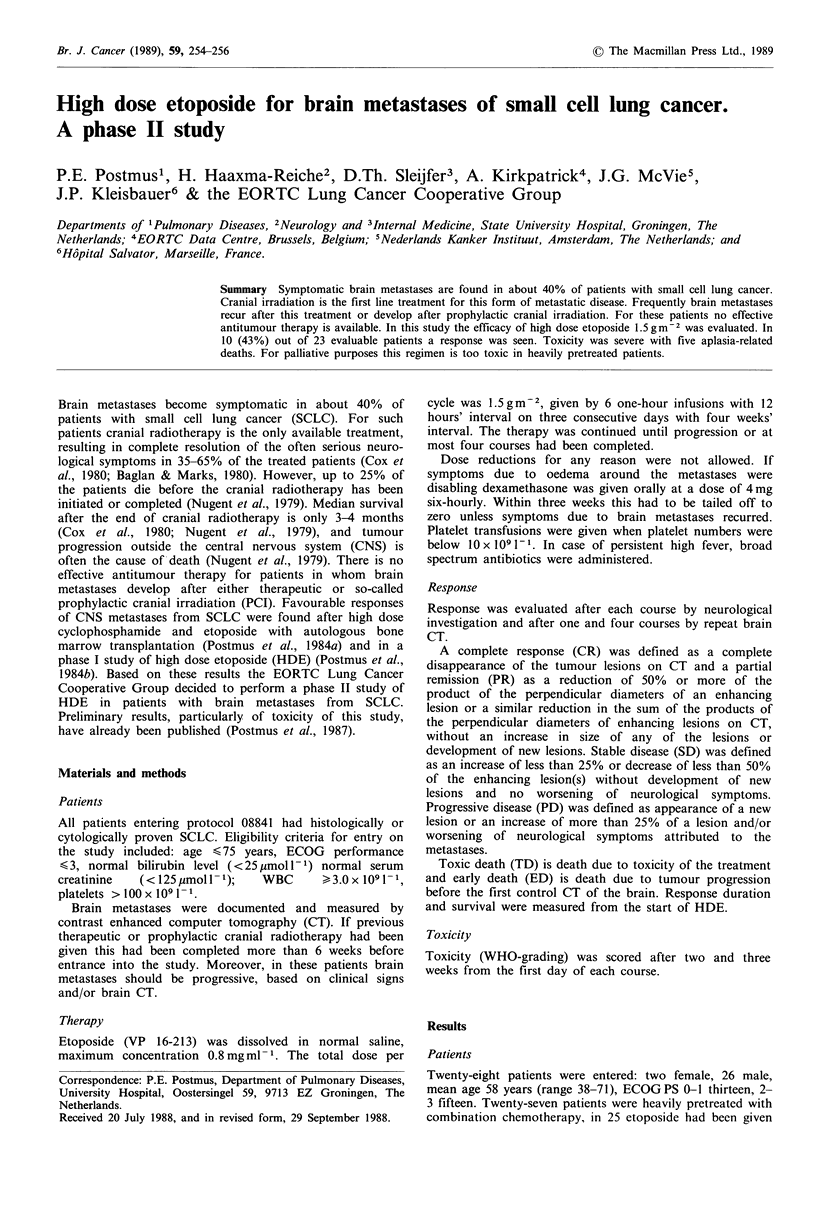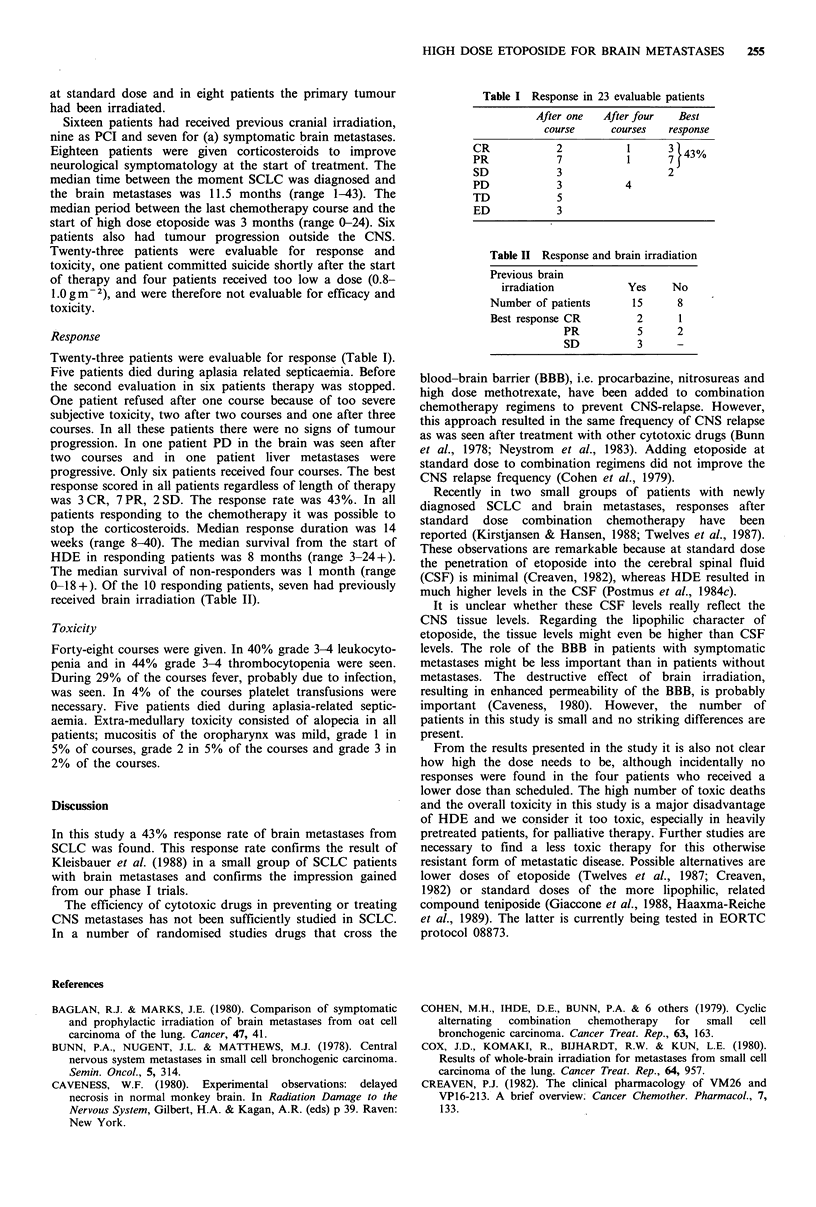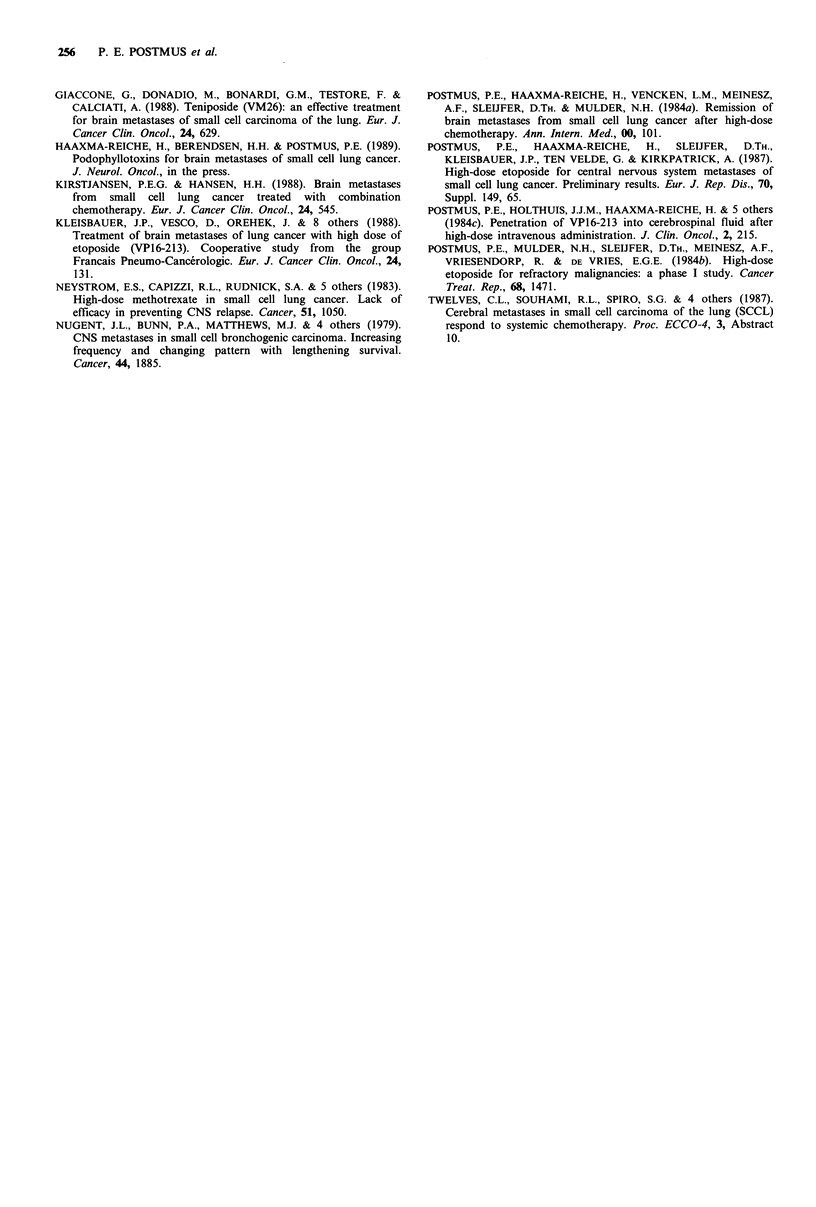# High dose etoposide for brain metastases of small cell lung cancer. A phase II study. The EORTC Lung Cancer Cooperative Group.

**DOI:** 10.1038/bjc.1989.52

**Published:** 1989-02

**Authors:** P. E. Postmus, H. Haaxma-Reiche, D. T. Sleijfer, A. Kirkpatrick, J. G. McVie, J. P. Kleisbauer

**Affiliations:** Department of Pulmonary Diseases, State University Hospital, Groningen, The Netherlands.

## Abstract

Symptomatic brain metastases are found in about 40% of patients with small cell lung cancer. Cranial irradiation is the first line treatment for this form of metastatic disease. Frequently brain metastases recur after this treatment or develop after prophylactic cranial irradiation. For these patients no effective antitumour therapy is available. In this study the efficacy of high dose etoposide 1.5 g m-2 was evaluated. In 10 (43%) out of 23 evaluable patients a response was seen. Toxicity was severe with five aplasia-related deaths. For palliative purposes this regimen is too toxic in heavily pretreated patients.


*Br. J. Cancer* (1989), **59**, 254–256 © The Macmillan Press Ltd., 1989

### High dose etoposide for brain metastases of small cell lung cancer. A phase II study

P.E. Postmus[1], H. Haaxma-Reiche[2], D.Th. Sleijfer[3], A. Kirkpatrick[4], J.G. McVie[5], J.P. Kleisbauer[6] & the EORTC Lung Cancer Cooperative Group

*Departments of [1]Pulmonary Diseases, [2]Neurology and [3]Internal Medicine, State University Hospital, Groningen, The Netherlands; [4]EORTC Data Centre, Brussels, Belgium; [5]Nederlands Kanker Instituut, Amsterdam, The Netherlands; and [6]Hôpital Salvator, Marseille, France.*

**Summary** Symptomatic brain metastases are found in about 40% of patients with small cell lung cancer. Cranial irradiation is the first line treatment for this form of metastatic disease. Frequently brain metastases recur after this treatment or develop after prophylactic cranial irradiation. For these patients no effective antitumour therapy is available. In this study the efficacy of high dose etoposide 1.5 $g\,m^{-2}$ was evaluated. In 10 (43%) out of 23 evaluable patients a response was seen. Toxicity was severe with five aplasia-related deaths. For palliative purposes this regimen is too toxic in heavily pretreated patients.

Brain metastases become symptomatic in about 40% of patients with small cell lung cancer (SCLC). For such patients cranial radiotherapy is the only available treatment, resulting in complete resolution of the often serious neurological symptoms in 35–65% of the treated patients (Cox *et al.*, 1980; Baglan & Marks, 1980). However, up to 25% of the patients die before the cranial radiotherapy has been initiated or completed (Nugent *et al.*, 1979). Median survival after the end of cranial radiotherapy is only 3–4 months (Cox *et al.*, 1980; Nugent *et al.*, 1979), and tumour progression outside the central nervous system (CNS) is often the cause of death (Nugent *et al.*, 1979). There is no effective antitumour therapy for patients in whom brain metastases develop after either therapeutic or so-called prophylactic cranial irradiation (PCI). Favourable responses of CNS metastases from SCLC were found after high dose cyclophosphamide and etoposide with autologous bone marrow transplantation (Postmus *et al.*, 1984*a*) and in a phase I study of high dose etoposide (HDE) (Postmus *et al.*, 1984*b*). Based on these results the EORTC Lung Cancer Cooperative Group decided to perform a phase II study of HDE in patients with brain metastases from SCLC. Preliminary results, particularly of toxicity of this study, have already been published (Postmus *et al.*, 1987).

### Materials and methods

### *Patients*

All patients entering protocol 08841 had histologically or cytologically proven SCLC. Eligibility criteria for entry on the study included: age $\leqslant 75$ years, ECOG performance $\leqslant 3$, normal bilirubin level ($<25\,\mu mol\,l^{-1}$) normal serum creatinine ($<125\,\mu mol\,l^{-1}$); WBC $\geqslant 3.0\times10^9\,l^{-1}$, platelets $>100\times10^9\,l^{-1}$.

Brain metastases were documented and measured by contrast enhanced computer tomography (CT). If previous therapeutic or prophylactic cranial radiotherapy had been given this had been completed more than 6 weeks before entrance into the study. Moreover, in these patients brain metastases should be progressive, based on clinical signs and/or brain CT.

### *Therapy*

Etoposide (VP 16-213) was dissolved in normal saline, maximum concentration $0.8\,mg\,ml^{-1}$. The total dose per cycle was $1.5\,g\,m^{-2}$, given by 6 one-hour infusions with 12 hours' interval on three consecutive days with four weeks' interval. The therapy was continued until progression or at most four courses had been completed.

Dose reductions for any reason were not allowed. If symptoms due to oedema around the metastases were disabling dexamethasone was given orally at a dose of 4 mg six-hourly. Within three weeks this had to be tailed off to zero unless symptoms due to brain metastases recurred. Platelet transfusions were given when platelet numbers were below $10\times10^9\,l^{-1}$. In case of persistent high fever, broad spectrum antibiotics were administered.

### *Response*

Response was evaluated after each course by neurological investigation and after one and four courses by repeat brain CT.

A complete response (CR) was defined as a complete disappearance of the tumour lesions on CT and a partial remission (PR) as a reduction of 50% or more of the product of the perpendicular diameters of an enhancing lesion or a similar reduction in the sum of the products of the perpendicular diameters of enhancing lesions on CT, without an increase in size of any of the lesions or development of new lesions. Stable disease (SD) was defined as an increase of less than 25% or decrease of less than 50% of the enhancing lesion(s) without development of new lesions and no worsening of neurological symptoms. Progressive disease (PD) was defined as appearance of a new lesion or an increase of more than 25% of a lesion and/or worsening of neurological symptoms attributed to the metastases.

Toxic death (TD) is death due to toxicity of the treatment and early death (ED) is death due to tumour progression before the first control CT of the brain. Response duration and survival were measured from the start of HDE.

### *Toxicity*

Toxicity (WHO-grading) was scored after two and three weeks from the first day of each course.

### Results

### *Patients*

Twenty-eight patients were entered: two female, 26 male, mean age 58 years (range 38–71), ECOG PS 0–1 thirteen, 2–3 fifteen. Twenty-seven patients were heavily pretreated with combination chemotherapy, in 25 etoposide had been given

Correspondence: P.E. Postmus, Department of Pulmonary Diseases, University Hospital, Oostersingel 59, 9713 EZ Groningen, The Netherlands.
Received 20 July 1988, and in revised form, 29 September 1988.

at standard dose and in eight patients the primary tumour had been irradiated.

Sixteen patients had received previous cranial irradiation, nine as PCI and seven for (a) symptomatic brain metastases. Eighteen patients were given corticosteroids to improve neurological symptomatology at the start of treatment. The median time between the moment SCLC was diagnosed and the brain metastases was 11.5 months (range 1–43). The median period between the last chemotherapy course and the start of high dose etoposide was 3 months (range 0–24). Six patients also had tumour progression outside the CNS. Twenty-three patients were evaluable for response and toxicity, one patient committed suicide shortly after the start of therapy and four patients received too low a dose (0.8–1.0 $g m^{-2}$), and were therefore not evaluable for efficacy and toxicity.

*Response*

Twenty-three patients were evaluable for response (Table I). Five patients died during aplasia related septicaemia. Before the second evaluation in six patients therapy was stopped. One patient refused after one course because of too severe subjective toxicity, two after two courses and one after three courses. In all these patients there were no signs of tumour progression. In one patient PD in the brain was seen after two courses and in one patient liver metastases were progressive. Only six patients received four courses. The best response scored in all patients regardless of length of therapy was 3 CR, 7 PR, 2 SD. The response rate was 43%. In all patients responding to the chemotherapy it was possible to stop the corticosteroids. Median response duration was 14 weeks (range 8–40). The median survival from the start of HDE in responding patients was 8 months (range 3–24+). The median survival of non-responders was 1 month (range 0–18+). Of the 10 responding patients, seven had previously received brain irradiation (Table II).

*Toxicity*

Forty-eight courses were given. In 40% grade 3–4 leukocytopenia and in 44% grade 3–4 thrombocytopenia were seen. During 29% of the courses fever, probably due to infection, was seen. In 4% of the courses platelet transfusions were necessary. Five patients died during aplasia-related septicaemia. Extra-medullary toxicity consisted of alopecia in all patients; mucositis of the oropharynx was mild, grade 1 in 5% of courses, grade 2 in 5% of the courses and grade 3 in 2% of the courses.

**Table I** Response in 23 evaluable patients

| | *After one course* | *After four courses* | *Best response* |
|---|---|---|---|
| CR | 2 | 1 | 3 } 43% |
| PR | 7 | 1 | 7 } 43% |
| SD | 3 | | 2 |
| PD | 3 | 4 | |
| TD | 5 | | |
| ED | 3 | | |

**Table II** Response and brain irradiation

| Previous brain irradiation | Yes | No |
|---|---|---|
| Number of patients | 15 | 8 |
| Best response CR | 2 | 1 |
| PR | 5 | 2 |
| SD | 3 | – |

### Discussion

In this study a 43% response rate of brain metastases from SCLC was found. This response rate confirms the result of Kleisbauer *et al.* (1988) in a small group of SCLC patients with brain metastases and confirms the impression gained from our phase I trials.

The efficiency of cytotoxic drugs in preventing or treating CNS metastases has not been sufficiently studied in SCLC. In a number of randomised studies drugs that cross the blood–brain barrier (BBB), i.e. procarbazine, nitrosureas and high dose methotrexate, have been added to combination chemotherapy regimens to prevent CNS-relapse. However, this approach resulted in the same frequency of CNS relapse as was seen after treatment with other cytotoxic drugs (Bunn *et al.*, 1978; Neystrom *et al.*, 1983). Adding etoposide at standard dose to combination regimens did not improve the CNS relapse frequency (Cohen *et al.*, 1979).

Recently in two small groups of patients with newly diagnosed SCLC and brain metastases, responses after standard dose combination chemotherapy have been reported (Kirstjansen & Hansen, 1988; Twelves *et al.*, 1987). These observations are remarkable because at standard dose the penetration of etoposide into the cerebral spinal fluid (CSF) is minimal (Creaven, 1982), whereas HDE resulted in much higher levels in the CSF (Postmus *et al.*, 1984*c*).

It is unclear whether these CSF levels really reflect the CNS tissue levels. Regarding the lipophilic character of etoposide, the tissue levels might even be higher than CSF levels. The role of the BBB in patients with symptomatic metastases might be less important than in patients without metastases. The destructive effect of brain irradiation, resulting in enhanced permeability of the BBB, is probably important (Caveness, 1980). However, the number of patients in this study is small and no striking differences are present.

From the results presented in the study it is also not clear how high the dose needs to be, although incidentally no responses were found in the four patients who received a lower dose than scheduled. The high number of toxic deaths and the overall toxicity in this study is a major disadvantage of HDE and we consider it too toxic, especially in heavily pretreated patients, for palliative therapy. Further studies are necessary to find a less toxic therapy for this otherwise resistant form of metastatic disease. Possible alternatives are lower doses of etoposide (Twelves *et al.*, 1987; Creaven, 1982) or standard doses of the more lipophilic, related compound teniposide (Giaccone *et al.*, 1988, Haaxma-Reiche *et al.*, 1989). The latter is currently being tested in EORTC protocol 08873.

### References

BAGLAN, R.J. & MARKS, J.E. (1980). Comparison of symptomatic and prophylactic irradiation of brain metastases from oat cell carcinoma of the lung. *Cancer*, **47**, 41.

BUNN, P.A., NUGENT, J.L. & MATTHEWS, M.J. (1978). Central nervous system metastases in small cell bronchogenic carcinoma. *Semin. Oncol.*, **5**, 314.

CAVENESS, W.F. (1980). Experimental observations: delayed necrosis in normal monkey brain. In *Radiation Damage to the Nervous System*, Gilbert, H.A. & Kagan, A.R. (eds) p 39. Raven: New York.

COHEN, M.H., IHDE, D.E., BUNN, P.A. & 6 others (1979). Cyclic alternating combination chemotherapy for small cell bronchogenic carcinoma. *Cancer Treat. Rep.*, **63**, 163.

COX, J.D., KOMAKI, R., BIJHARDT, R.W. & KUN, L.E. (1980). Results of whole-brain irradiation for metastases from small cell carcinoma of the lung. *Cancer Treat. Rep.*, **64**, 957.

CREAVEN, P.J. (1982). The clinical pharmacology of VM26 and VP16-213. A brief overview. *Cancer Chemother. Pharmacol.*, **7**, 133.

GIACCONE, G., DONADIO, M., BONARDI, G.M., TESTORE, F. & CALCIATI, A. (1988). Teniposide (VM26): an effective treatment for brain metastases of small cell carcinoma of the lung. *Eur. J. Cancer Clin. Oncol.*, **24**, 629.

HAAXMA-REICHE, H., BERENDSEN, H.H. & POSTMUS, P.E. (1989). Podophyllotoxins for brain metastases of small cell lung cancer. *J. Neurol. Oncol.*, in the press.

KIRSTJANSEN, P.E.G. & HANSEN, H.H. (1988). Brain metastases from small cell lung cancer treated with combination chemotherapy. *Eur. J. Cancer Clin. Oncol.*, **24**, 545.

KLEISBAUER, J.P., VESCO, D., OREHEK, J. & 8 others (1988). Treatment of brain metastases of lung cancer with high dose of etoposide (VP16-213). Cooperative study from the group Francais Pneumo-Cancérologic. *Eur. J. Cancer Clin. Oncol.*, **24**, 131.

NEYSTROM, E.S., CAPIZZI, R.L., RUDNICK, S.A. & 5 others (1983). High-dose methotrexate in small cell lung cancer. Lack of efficacy in preventing CNS relapse. *Cancer*, **51**, 1050.

NUGENT, J.L., BUNN, P.A., MATTHEWS, M.J. & 4 others (1979). CNS metastases in small cell bronchogenic carcinoma. Increasing frequency and changing pattern with lengthening survival. *Cancer*, **44**, 1885.

POSTMUS, P.E., HAAXMA-REICHE, H., VENCKEN, L.M., MEINESZ, A.F., SLEIJFER, D.TH. & MULDER, N.H. (1984*a*). Remission of brain metastases from small cell lung cancer after high-dose chemotherapy. *Ann. Intern. Med.*, **00**, 101.

POSTMUS, P.E., HAAXMA-REICHE, H., SLEIJFER, D.TH., KLEISBAUER, J.P., TEN VELDE, G. & KIRKPATRICK, A. (1987). High-dose etoposide for central nervous system metastases of small cell lung cancer. Preliminary results. *Eur. J. Rep. Dis.*, **70**, Suppl. 149, 65.

POSTMUS, P.E., HOLTHUIS, J.J.M., HAAXMA-REICHE, H. & 5 others (1984*c*). Penetration of VP16-213 into cerebrospinal fluid after high-dose intravenous administration. *J. Clin. Oncol.*, **2**, 215.

POSTMUS, P.E., MULDER, N.H., SLEIJFER, D.TH., MEINESZ, A.F., VRIESENDORP, R. & DE VRIES, E.G.E. (1984*b*). High-dose etoposide for refractory malignancies: a phase I study. *Cancer Treat. Rep.*, **68**, 1471.

TWELVES, C.L., SOUHAMI, R.L., SPIRO, S.G. & 4 others (1987). Cerebral metastases in small cell carcinoma of the lung (SCCL) respond to systemic chemotherapy. *Proc. ECCO-4*, **3**, Abstract 10.